# Self-Sensing Composites: *In-Situ* Detection of Fibre Fracture

**DOI:** 10.3390/s16050615

**Published:** 2016-04-28

**Authors:** Shoaib A. Malik, Liwei Wang, Paul T. Curtis, Gerard F. Fernando

**Affiliations:** 1 School of Metallurgy and Materials, University of Birmingham, Birmingham B15 2TT, UK; shoaibamalik@yahoo.com (S.A.M.); wlw@mju.edu.cn (L.W.); 2 Physical Sciences Department, Dstl Porton Down, Salisbury, Wilts SP4 0JQ, UK; ptcurtis@taz.dstl.gov.uk

**Keywords:** self-sensing, optical fibre sensors, fibre fracture, damage detection, structural health monitoring

## Abstract

The primary load-bearing component in a composite material is the reinforcing fibres. This paper reports on a technique to study the fracture of individual reinforcing fibres or filaments in real-time. Custom-made small-diameter optical fibres with a diameter of 12 (±2) micrometres were used to detect the fracture of individual filaments during tensile loading of unreinforced bundles and composites. The unimpregnated bundles were end-tabbed and tensile tested to failure. A simple technique based on resin-infusion was developed to manufacture composites with a negligible void content. In both cases, optical fibre connectors were attached to the ends of the small-diameter optical fibre bundles to enable light to be coupled into the bundle via one end whilst the opposite end was photographed using a high-speed camera. The feasibility of detecting the fracture of each of the filaments in the bundle and composite was demonstrated. The *in-situ* damage detection technique was also applied to E-glass bundles and composites; this will be reported in a subsequent publication.

## 1. Introduction

A number of techniques have been developed and deployed for detecting damage in fibre reinforced composites. For example, dye penetrant enhanced x-ray radiography has been used to map the distribution of matrix cracks subsequent to mechanical loading [1]. Stereo radiography has also been demonstrated as a viable technique to obtain perception of the depth with regard to matrix damage in composite materials [2,3]. Advances in x-ray tomography currently permits 3-dimensional mapping of the distribution of voids, fibre and matrix damage [4]. Edge-replication, involving impressing a solvent-loaded cellulose acetate strip on the edge of a polished composite, during mechanical loading, has been used to monitor the progression of matrix cracking and delaminations [5]. The advent of low-cost charged coupled device (CCD) microscopes have enabled real-time monitoring of damage evolution from polished edges of composites.

The detection of delaminations in composites continues to attract significant attention. The “coin-tap” test involves a coin that is gently tapped and traversed on the surface of the composite by a skilled operator. The changes in the tactile feedback and the general acoustic characteristic are used to infer the presence of delaminations. This technique has progressed to the use of specified transducers to record the vibration per mechanical impulse on the surface of the composite [6]. Ultrasonic C-scan-based inspection is a popular technique to detect delaminations [7]. Since this technique requires the sample to be immersed or covered with a liquid, air-coupled transducers have been developed [8]. Laser shearography has been shown to be effective for detecting debonding and delaminations in the surface layers of composites [9,10,11]. Moire interferometry [12,13] and digital image correlation [14] are effective techniques for monitoring surface deformation and strain [15]. Thermography is a practical technique for detecting temperature rises in the sample during dynamic loading to monitor the evolution of damage in composites [16]; this can involve the use of surface-mounted temperature sensors [17] or infrared cameras [18]. Advances in this technique include pulsed [19] and lock-in thermography [20]. Acoustic emission is a technique where the acoustic emissions emanating during the mechanical loading of a composite is monitored using piezo-electric transducers [21]. A variation on this theme is acousto-ultrasonic [22,23]. Finally, it is reasonable to state that visual inspection will continue to be used to infer specified forms of damage in composites [24].

Whilst the above-mentioned techniques are mature with regard to damage detection in composites, the quest for *in situ* techniques for assessing the structural integrity of composites continues to attract significant attention. For example, the electrical [25] and optical [26] properties of carbon and glass fibres respectively, have been demonstrated as practical techniques for detecting fibre fractures in composites.

The advantages of optical fibre-base sensor systems include [27]: (i) the circular cross-section of optical fibres with diameters in the range 20–200 micrometres. This makes it relatively easy to embed them within the preforms at the time of manufacturing [28]; (ii) the surface chemistry of optical fibres is receptive to conventional surface treatments such as silane coupling agents [29]; (iii) they are immune from electromagnetic interference, hence, unlike their electrical counterparts, they can be used in environments with high electrical potentials; (iv) a unique characteristic is the possibility of monitoring multiple parameters on a single optical fibre [30]; (v) distributed sensing is also a practical reality [31]; and (vi) a wide range of optical fibre types are available commercially, thus appropriate compositions can be selected to suit specified end-use applications [32].

For example, fibre-optic sensor systems have been developed to enable the following measurands to be accessed in real-time in composites: displacement [33], strain and temperature [34], vibration [35], pressure [36,37], acoustic emission [38], refractive index [39], quantitative data on the relative concentrations of specified chemical species during cross-linking [40], diffusion of moisture [41], fatigue [42] and impact damage [43].

With reference to the in-service deployment of sensors, trailing inter-connecting electrical wires and optical fibres are generally not a practical proposition. Hence, various sensor protection systems and data communication strategies have been developed to overcome this basic problem including: (a) wireless devices [44]; (b) free-space optical transmission [45]; (c) inductive coupling of electrical signals; (d) through-thickness transmission of light [46]; and (e) the use of connectors on the surface of the structure [32,47].

This current paper reports on a new approach to enable *in situ* condition monitoring of glass fibre reinforced composites. The feasibility of using conventional E-glass fibres for damage detection [48] and process monitoring [49] was demonstrated previously. In the current case, custom-made small-diameter optical fibres, with an equivalent diameter to 2400 Tex E-glass, were used to develop a methodology to study in real-time, the fracture of individual filaments in un-impregnated bundles and composites during tensile loading.

## 2. Materials and Methods

### 2.1. Materials

#### 2.1.1. Matrix

The primary resin system used was a two-part epoxy/amine, EPO-TEK^®^-314 (Promatech Ltd., Cirencester, UK) with a stoichiometric ratio of 100:6 for the epoxy and amine respectively. The cross-linking schedule was 3-h at 120 °C. The refractive index of the mixed resin system was measured on an Abbe refractometer and was found to be 1.496 (589.6 nm and 20 °C). The tensile strength and the failure strain of cast dog-bone test specimens of neat resin were 27.2 MPa and 1.1%, respectively.

#### 2.1.2. Fibres

**Figure 1:**
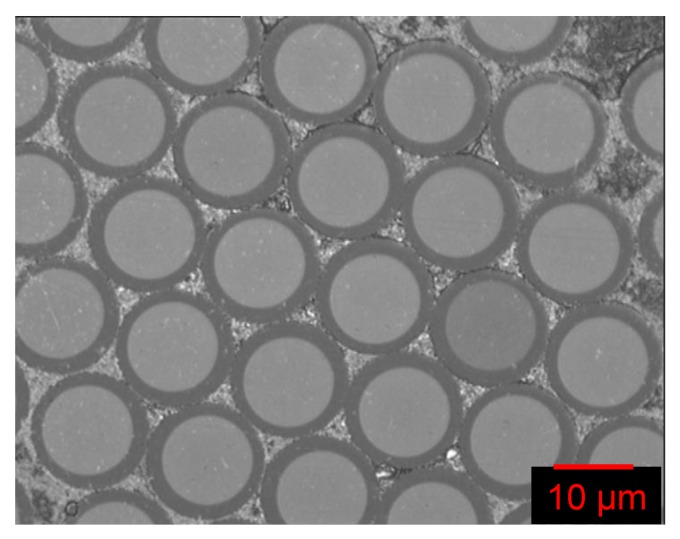
Micrograph of the custom-made small-diameter optical fibres showing the core and the cladding.

Custom-made small-diameter optical fibres (SDOF) were sourced from Aomolin Ltd. (Beijing, China). The average diameter of the SDOF was 12 (±2) µm. The composition of the SDOF is similar to F2 glass and each fibre bundle consisted of approximately 2800 (±200) individual filaments. Figure 1 shows a magnified transverse section of the SDOFs where the presence of the core and cladding can be seen.

### 2.2. Sample Preparation

#### 2.2.1. Fibre Bundles

In order to enable efficient coupling of light in and out of the fibre bundle, sub-miniature adapters (SMA) Type-A (ID-11275A, Thorlabs, Exeter, UK) were used as connectors at the ends of the bundle. The standard SMA connectors were supplied with a bore diameter of 1.2 mm; in the current study they were drilled and deburred to 1.4 mm to accommodate the as-received SDOF fibre bundles. Prior to potting the ends of the fibre bundles in the SMA connectors, they were immersed in distilled water and placed in an ultrasonic bath for 20-minutes. The SMA were then dried in an air-circulating oven (Memmert, Schwabach, Germany) at 80 °C for 2 h and stored in a desiccator until required.

The required length of the SDOF was cut from the spool using a fresh portion of a razor blade. The ends of the SDOF bundle were potted in the SMA connector using an optical-grade adhesive (Opti-tec 5007, Intertronics, Kidlington, UK). The cross-linking schedule for the potting resin was 24-h at 25 °C. The refractive index of the (mixed) potting resin, measured on an Abbe refractometer, was 1.550.

The fibre bundles with the SMA connectors were polished using an automatic optical fibre polisher (APC-8000, Senko, Aldermaston, UK). One of the SMA connectors with the polished SDOF bundle was imaged during the tensile tests using a high-speed CCD camera (FastCAM 1024 PCI, Photron, West Wycombe, UK) whilst the opposite end was illuminated using a white-light source (Intralux-4000, Warner Instruments, Hamden, CT, USA).

#### 2.2.2. Production of SDOF Composites

**Figure 2:**
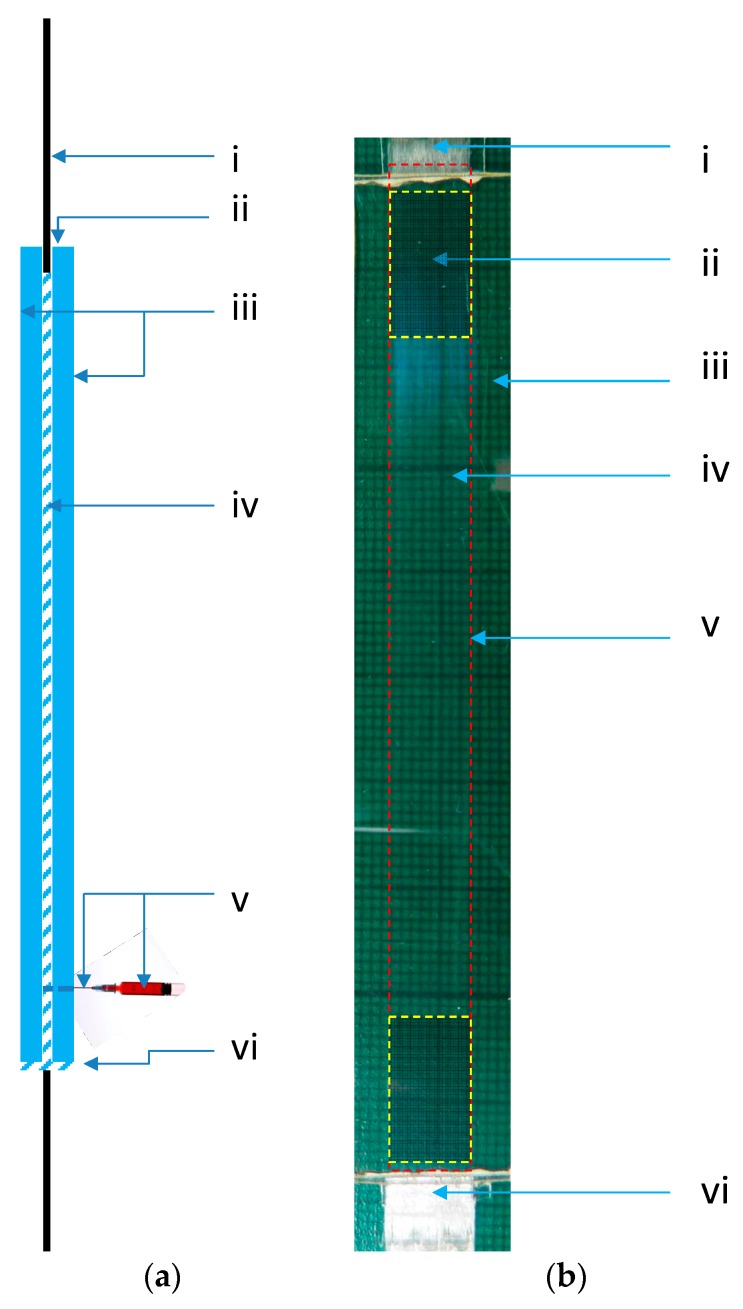
(**a**) Schematic illustration of the glass mould assembly that was used to manufacture the SDOF composites. (i) Un-impregnated SDOF bundle. (ii) Unsealed top of the glass mould. (iii) Pair of glass plates with spacers (the spacers are not shown). (iv) Silicone sealant used to seal the sides and the bottom of the mould. (v) Syringe and needle assembly. (vi) Sealed bottom of the glass mould; (**b**) A representative photograph of a SDOF bundles self-sensing composite samples manufactured by the resin-injection technique. The coded items are as follows: (i) un-impregnated SDOF; (ii) The location of the intended aluminium end-tab; (iii) Neat resin; (iv) SDOF composite; (v) The dotted larger rectangle represents the boundary of composite (impregnated and cured); (vi) Un-impregnated SDOF bundle.

A schematic illustration of the glass mould that was used to fabricate the SDOF composites is shown in Figure 2a. With reference to Figure 2a, a glass plate was secured on a metal plate of dimension 50 × 50 cm which was covered with an-adhesive-backed PTFE sheet (Aerovac, Keighley, UK). A SDOF bundle was secured on the bottom glass plate using small bull-dog clips at the ends of the bundle. This restricted any gross movement of the SDOF bundle.

A thin layer (≈0.1 mm) of a silicone resin (RTV-3140-Dow Corning, Univar Specialty Consumables, Tamworth, UK) was applied on the PTFE spacers that were attached to the bottom glass plate. A needle (part of a syringe assembly) was also secured to the bottom glass plate, before the top glass plate was positioned and aligned with the lower glass plate. The glass plates were clamped and sealed along three edges using the silicone sealant; the top of the mould was not sealed. The sealant was then left to cure for 24-h at ambient temperature.

Five grams of the EPO-TEK^®^ 314 resin system per sample was mixed thoroughly and then degassed in a vacuum chamber at −15 mm of Hg for 20 min. The degassed resin was injected gradually via a syringe (MS403-10, Thorlabs, Exeter, UK) until the mould was filled. A semi-automated syringe-pump (Alladin-220, World Precision Instruments, Hitchin, UK) was used to drive the plunger on the syringe assembly that contained the resin. The mould assembly was left in a vertical position for 30 min to ensure that the fibres were impregnated thoroughly. The syringe was removed and the entry point of the needle located outside the mould was sealed with a silicone sealant. The assembly was placed in an air-circulating oven (Memmert) and the resin system was cured at 120 °C for 3 h. After cooling to room temperature, the composite was removed from the mould and the needle was detached. The dimensions of the rectangular composite specimens were 150 mm (length) × 20 mm (width) × 0.8 mm (thickness). The gauge length of the composite was similar to that of the SDOF fibre bundle (100 mm).

#### 2.2.3. End-Tabbing

**Figure 3:**
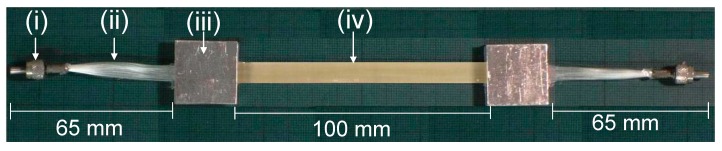
Photograph of a self-sensing composite with SMA connectors and end-tabs: (i) SMA connector; (ii) un-impregnated section of the SDOF bundle; (iii) end-tab; and (iv) void-free composite.

Conventional procedures were used to bond aluminium end-tabs to the ends of the bundles and composites. The adhesive used for end-tabbing was Scotch-Weld 9323 (3M, Distributor: Viking Industrial, Keighley, UK). A photograph of an end-tabbed SDOF composite with a pair of SMA connectors is shown in Figure 3.

### 2.3. Tensile Testing

Tensile testing of the SDOF bundles and composites were performed on an Instron model-5566 mechanical test machine (Instron, High Wycombe, UK). The extension and load/time traces were recorded using a data acquisition system and proprietary software. The machine was instrumented with a 10 kN load-cell where the accuracy of the applied load was ±0.5%. The tensile tests were carried out at a cross-head displacement rate of 1 mm/minute at 20 °C in a temperature regulated laboratory (±2 °C).

### 2.4. Monitoring the Transmitted Light Intensity

**Figure 4:**
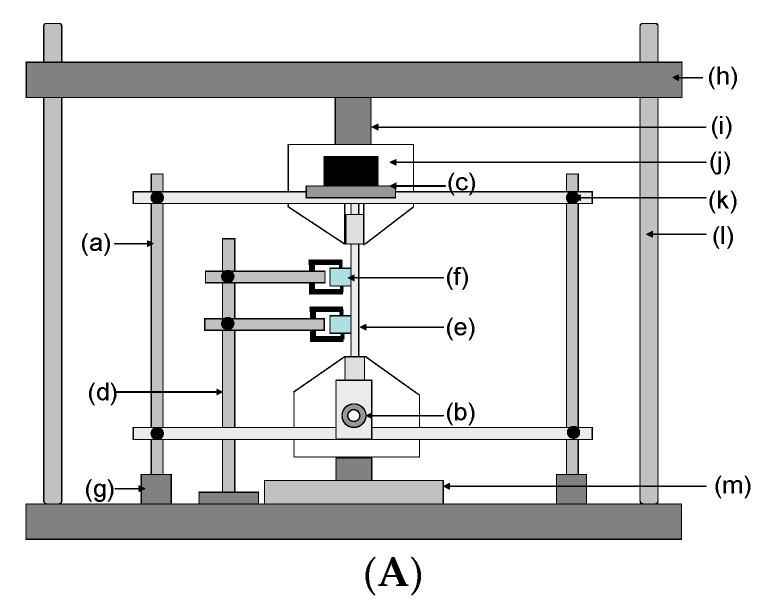

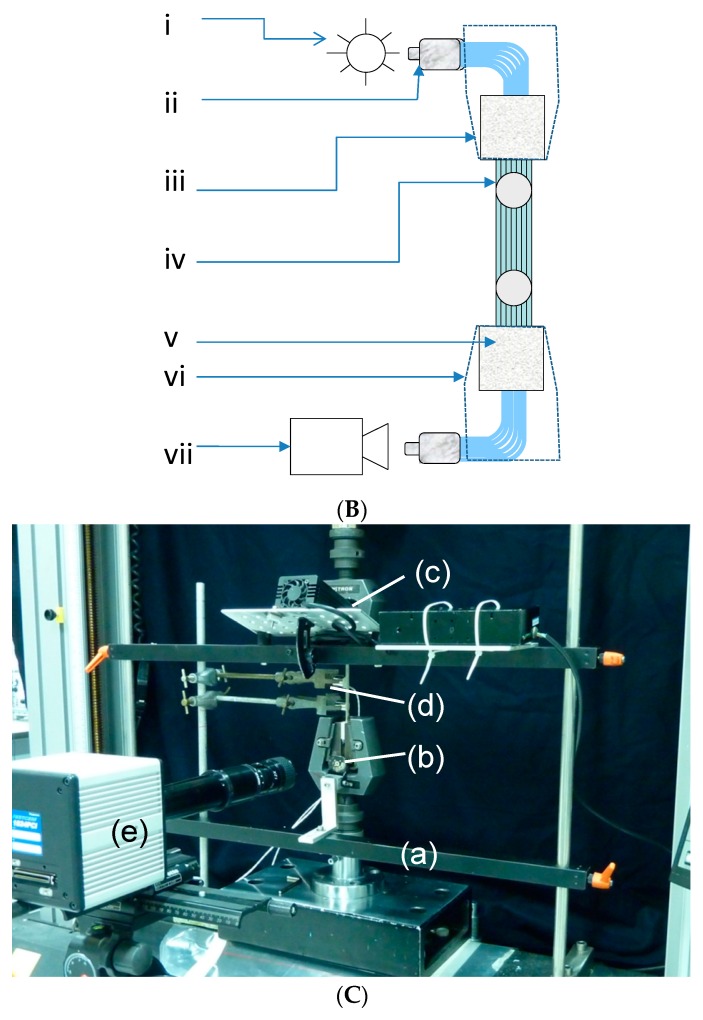
(**A**) Schematic illustration for the tensile test experimental setup: (a) jig that housed the light source and SMA connectors in position; (b) attachment for the SMA connector (high-speed camera-end); (c) platform for the light source; (d) laboratory retort stand for securing the piezoelectric transducers; (e) tensile test specimen; (f) Piezo-electric transducer held in place using a retort stand; (g) detachable base for the jig; (h) moving-end of the Instron tensile test machine; (i) load-cell; (j) white-light source; (k) height adjustment mechanism for the jig; (l) Instron machine columns; and (m) base of the Instron machine (stationary-end); (**B**) Schematic illustration of the location of the light source, grips of the tensile testing machine, the piezo-electric transducers and the high-speed camera. (i) White light (when testing SDOF) or laser source (in conjunction with E-glass); (ii) SMA connector; (iii) Mobile-end of the jaws of the mechanical test machine; (iv) Piezo-electric transducer; (v) Aluminium end-tab; (vi) Stationary-end of the jaws of the mechanical test machine; (vii) High-speed CCD camera; (**C**) Photograph of the tensile test setup: (a) Tensile test fixture; (b) Attachment for the SMA connector (camera-end); (c) Attachment for the white light or laser-light source; (d) Piezo-electric transducers secured in place using a laboratory retort stand; (e) High-speed camera.

A schematic illustration of the experimental set-up for tensile testing the SDOF bundle or composite and the associated instrumentation is shown in Figure 4A. The end-tabbed specimen was clamped between the grips of the Model-5566 Instron machine and two piezo-electric transducers were secured in intimate contact with the test specimen. The primary function of the piezo-electric transducers was to trigger the high-speed camera when the first fibre fracture is detected. One of the SMA connectors was attached to the light source and other connector was fixed to a housing to enable the end of the bundle to be imaged via the high-speed camera.

A schematic representation of the relative positions of the light source, high-speed CCD camera and the piezo-electric transducers is illustrated in Figure 4B.

Figure 4C shows a photograph of the actual experimental set up that was used for tensile testing the SDOF bundle and composite.

### 2.5. Acoustic Emission Transducers

The AE transducers used in the study were two narrowband R15 sensors supplied by the Physical Acoustics. Coupling between the piezo-electric transducers and the specimen was Corporation enabled using a silicone gel (494-118, RS Components, Birmingham, UK). The piezoelectric transducer were connected to a pre-amplifier (model, 2/4/6) and integrated to a data acquisition unit (PCI-2, Physical Acoustics Corporation, Princeton, NJ, USA). Prior to tensile testing, the response of the piezoelectric transducer was assessed by performing a pencil lead-break test. This test involved fracturing a 0.2 mm pencil lead (2B) at approximately 5 mm on the lower end-tab. The amplitude of this signal was recorded. A threshold setting of 40 dB was used for all the tensile tests.

### 2.6. High-Speed Charge-Coupled Device Camera

A FastCAM 1024 PCI (Photron, West Wycombe, UK) high-speed charge-coupled device camera with a resolution of 1024 × 1024 pixels was used to image one end of the SDOF test specimen whilst the opposite end was illuminated. It was necessary to develop protocols to synchronise the various items of equipment such as the mechanical test machine, the acoustic emission transducers and the high-speed camera.

The first signal from the acoustic emission sensor was used to trigger the high-speed camera. In other words, when the acoustic emission transducer detected the first acoustic event during the tensile loading of the test specimens; this signal was used to trigger the recording of the high-speed camera. This was achieved using the transistor-transistor logic (TTL) input of the high-speed camera. The load and the strain data from the Instron’s data acquisition card were transferred to the data acquisition system on the piezoelectric acoustic emission system. The AE system provided the option for two parametric inputs; this was used to record two independent inputs (±10 V) signals through the parametric channels. The output voltage from the Instron’s data acquisition card was used as an input to the AE parametric channels. In this way, the load and extension were recorded with the AE events. 

### 2.7. Image Analysis

Image analysis was a key component of the current work as it was necessary to record and to extract information from images of polished SDOF bundle within SMA connector during tensile loading. The aim was to track the change in light intensity of the individual filaments then to relate it with other sources of information, namely acoustic emission and mechanical loading. The number of images taken by the high-speed camera ranged from 7000–8000 per test. Therefore, software routines were developed to enable image processing and analysis. Matlab™ (MathWorks, Cambridge, UK) was used to analyse the images taken by the high-speed camera. A macro was developed that analysed the first image and identified each filament in the bundle as an individual entity; the centroidal position of each fibre was specified using x and y-coordinates. For the remainder of the images obtained during tensile loading, the light intensity of each fibre was calculated using the same coordinate positions.

## 3. Results

### 3.1. Quality of the Test Specimens

With regard to the production of the SDOF bundles, due care and attention was given to ensure that the fibres within the end-tab regions were impregnated fully. Fibre slippage was not observed during any of the tensile tests. The main advantages of the resin injection technique for manufacturing SDOF composites are as follows: (i) it provided composites a ‘good’ surface finish and uniform thickness. The measured thickness of the specimens manufactured from this technique was found to be 0.75 ± 0.01 mm. The average fibre volume fraction determined using the image analysis method was 45% ± 2%; (ii) it was comparatively easy to control and align the fibres within the mould.

### 3.2. High-Speed Photography and Image Analysis

The number of images captured by the high-speed camera ranged from 7000 to 8000 per tensile test. Hence, appropriate software routines had to be developed to enable large-scale image processing and analysis. A macro was developed in Matlab™ to read the first image and to identify each fibre as an individual entity; the centroidal position of each fibre was specified using x and y-axis coordinates. The light intensity of each fibre was calculated using these coordinate positions using a 0–255 scale where ‘0’ represents no transmitted light and ‘255’ corresponds to bright white light. The coordinates for the rest of the images remained the same since the camera-end of fibre bundle was stationary.

**Figure 5:**
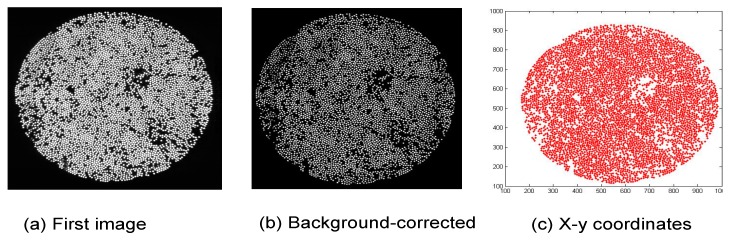
(**a**–**c**) Illustration of the analysis steps involved with processing the images obtained via the high-speed camera: (**a**) image from the high-speed camera at t = 0 (first image); (**b**) background-corrected image using Matlab™ ; and (**c**) x-y plot for the location of the individual fibres.

Figure 5a shows the first image that was captured at the start of the tensile test (t = 0) involving the SDOF bundle. Figure 5b represents the transposition of Figure 5a using the Matlab™ software routine. This step involved, background correction and adjustment of the luminance threshold. Figure 5c represents the x-y location plot after the manipulation in Matlab™ of the image shown in Figure 5a,b. The quality and processing of the images presented in Figure 5c was found to be adequate in order to enable the fracture of the SDOFs to be tracked during the tensile test. The Matlab™ routines were able to identify and process each individual fibre with an accuracy of 94% ± 2%. This was confirmed by counting the fibres manually in sections of the images and then comparing them to the number of fibres reported by the Matlab routines.

### 3.3. Transmitted Light Intensity

**Figure 6:**
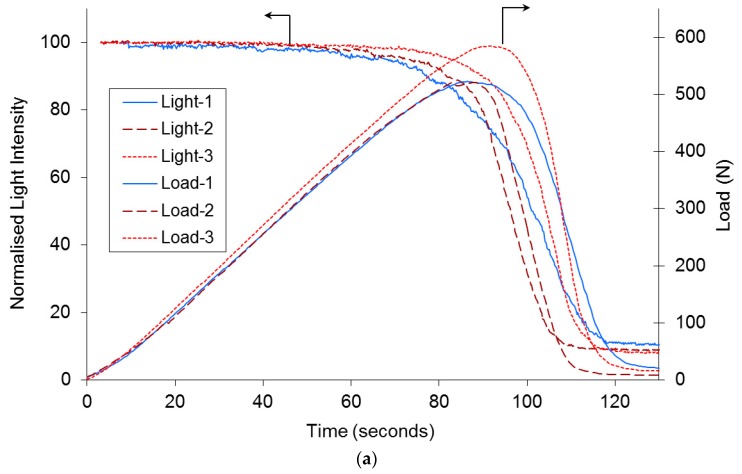

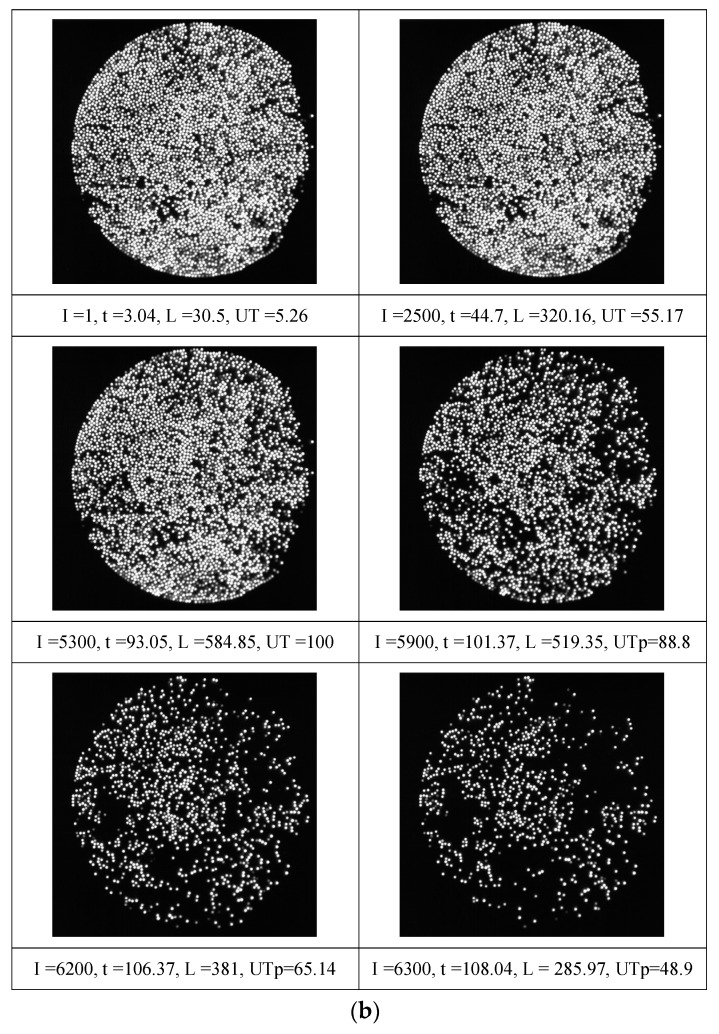
(**a**) Typical graph showing the normalised transmitted light intensity and applied load as a function of time for three different samples of the SDOF bundles; (**b**) Illustration of the fracture sequence of the filaments in the SDOF bundle.

Although the initial transmitted light intensity was found to vary by less than 5% from sample-to-sample, the data were normalised to the initial value prior to tensile loading. Typical normalised load/time and normalised light intensity/time traces for three individual SDOF bundles are shown in Figure 6a. With reference to Figure 6a, the following observations are made:

(i) The initial decrease in the transmitted light intensity for the three samples was 21%, 16% and 9%. This initial marginal decrease in the transmitted light intensity during tensile loading may be attributed to the failure of the weaker fibres in the bundle. Fibre-to-fibre contact may have also been responsible for the observed initial attenuation. Poisson’s contraction of the SDOF during tensile loading could also influence the light transmission characteristics via the stress optic coefficient. Some degree of lateral compression within the end-tab may have also occurred as the sample was gripped within the jaws of the mechanical test machine.
(ii) It is apparent in Figure 6a that the load *versus* extension plot does not exhibit a catastrophic failure mode for the SDOF bundle after the peak-load was attained. This may be attributed to one or more of the following. (a) Variable tension in the filaments: although due care and attention was taken whilst end-tabbing the bundles, it was not possible to guarantee that each of the filaments was under uniform tension; (b) Fibre alignment: Although the SDOF bundle did not contain any twists, it is conceivable that not all the filaments in the bundle were aligned parallel to the loading direction; (c) Strength distribution: Since the filaments in the bundle have a distribution of strength, the weaker filaments will fail first when loaded in tension; (d) Variable diameter: On inspecting Figure 1, it is seen that the diameters of the SDOFs were in the range 12 ± 1 micrometres.
(iii) An apparent correlation is seen between the time (x-axis) when there is deviation in the original slope and the peak load attained by the sample. In other words, in cases where the time for the deviation of the transmitted light intensity occurs earlier during tensile loading, the corresponding peak load attained by the sample is lower. In the current case, the deviation from the initial slope for the transmitted light intensity for samples 1, 2 and 3 is seen to take place at 58, 62 and 78 s respectively, and the corresponding peak loads attained were 510, 517 and 584 N respectively. However, this observation may also be due to the precise number of filaments present in the SDOF bundle. Correlation between the mechanical test data and the visualisation of the filament fracture processes are shown in Figure 6a,b.

Figure 6b represents the visualisation of the fracture behaviour of the filaments in the bundle recorded by the high-speed camera for the SDOF bundle. The abbreviations “I”, “t”, “L” “UT” and “UTp” correspond to image number, time (s), load (Newton), percentage of the peak-load, and percentage of peak-load after failure *i.e.*, after the peak-load was attained respectively. 

The first image was captured after 3.04 s when the first acoustic emission hit was detected by the piezo-electric transducers; this triggered the continuous recording of the high-speed camera. The images shown in Figure 6b were extracted (from the series of images recorded at 60 frames per second during the test) at approximately 40-s interval from the onset of continuous recording of the high-speed camera. The peak-load attained by this SDOF bundle was 584.5 N. The peak load was attained after 93.05 s at 5300th image, where the majority of fibres were still intact. After the peak load (5300th image), the next images, *i.e.*, 5900th, 6200th and 6300th were extracted at 8.32, 5, and 1.67 s respectively. This was undertaken to demonstrate the light attenuation characteristics before and after the peak-load was reached.

On inspecting the 1st and the 5300th image (the latter corresponding to an ultimate peak-load of 584.5 N), it is apparent that the majority of the filaments in the SDOF bundle are intact. This observation agrees with that seen in Figure 6a where the transmitted light intensity was relatively constant up to approximately 96% of the ultimate tensile strength. After the peak-load of 584.5 N is reached, an accelerated rate in the fracture of the filament was observed. The observed non-catastrophic failure of fibre bundles has been reported by previous researchers [43,48,50,51]. Acoustic emission has also been used to record the failure of individual filaments in the bundle.

**Figure 7:**
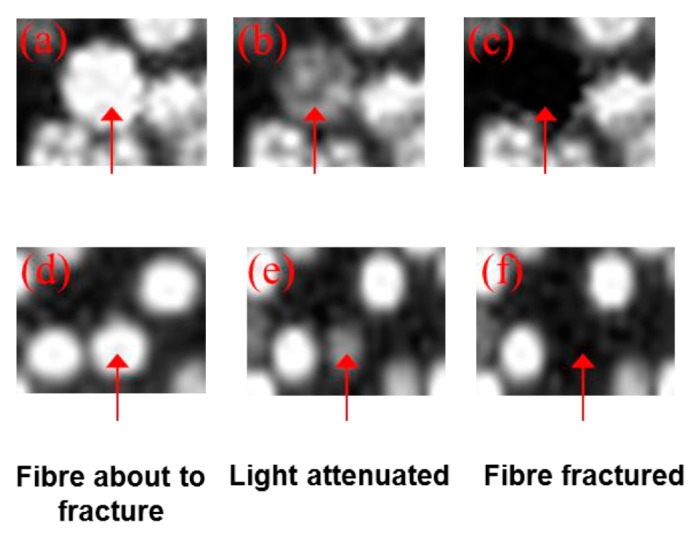
(**a**–**c**) Identification of imminent fibre fracture by monitoring the transmitted light intensity for a SDOF (**a**,**d**): whilst the sample was loaded in tension; (**b**,**e**) just prior to fracture; and (**c**,**f**) immediately after fibre fracture.

The discussion so far has demonstrated that it is possible to study in real-time the fracture of individual filaments of the small-diameter optical fibre bundles. An investigation was carried out to determine if it would be possible to detect the imminent fracture of an individual filament during tensile loading, *i.e.*, the instance just prior to catastrophic failure. Figure 7a–f show a typical set of sequential images captured at 60 frames per second during the tensile testing of a SDOF bundle; the images have been magnified digitally. The images shown in Figure 7a–c represent the transmitted light intensity through the highlighted filament just before, during and after fracture, respectively. On inspecting these images, it is readily apparent that the transmitted light intensity diminishes significantly as it is about to fracture; this is followed by total attenuation after the fracture of the filament.

This trend was observed for all the filaments. This is the first reporting of such capability to identify a filament just prior to fracture.

### 3.4. Tensile Testing of SDOF Composites

**Figure 8:**
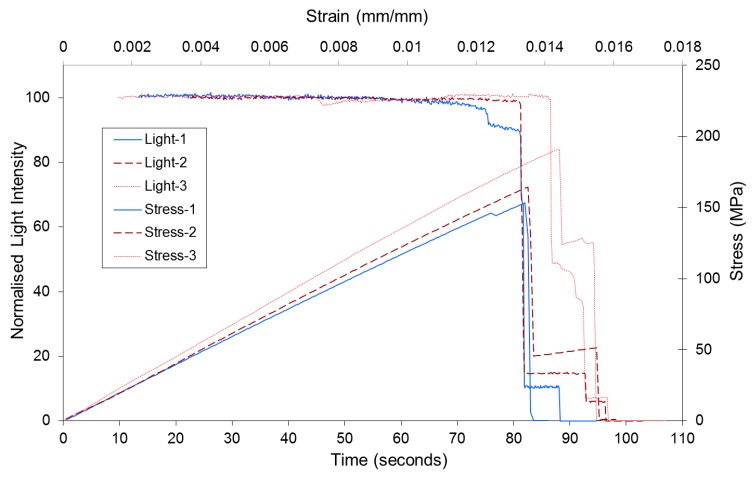
Representative graphs showing the normalised transmitted light intensity and applied stress as a function of time and strain for three SDOF composites.

Figure 8 shows typical normalised light intensity/time and stress/time graphs for three SDOF composite. It can be seen that the transmitted light intensity is relatively constant until approximately 90% of the ultimate failure stress. At the onset of catastrophic failure, the transmitted light intensity decreased dramatically. With reference to Figure 8, the drops in the normalised light intensity/time and the load/time plots correspond to the development of a longitudinal splitting and transverse fractures of a section of the composite; a corresponding intensity-drop is observed in the normalised intensity/time trace. In general, the characteristic features in the load/time traces were reproduced in the normalised light intensity/time data.

The average mechanical properties of the composites are summarised in Table 1. The stresses *versus* strain plots were linear until failure and the tensile test results were within 5%–8%. The average tensile strength and failure strain for the SDOF composites were 186.5 MPa and 1.4% respectively.

### 3.5. Image Analysis of SDOF Composites

**Figure 9:**
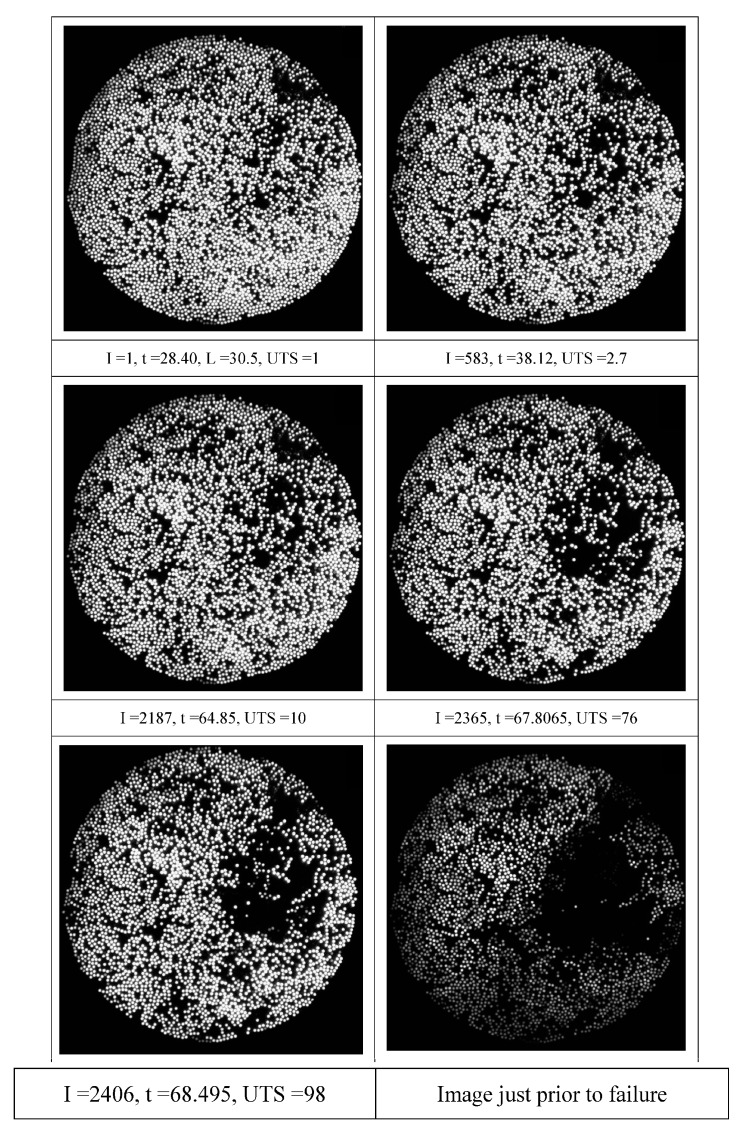
Summary of tensile test and *in-situ* monitoring of light attenuation characteristics for the SDOF composites.

Figure 9 represents a summary of relevant tensile test data and the corresponding transmitted light characteristics of a SDOF composite during tensile loading. With reference to Figure 9, the abbreviations “I”, “t”, “L” and “UT” corresponds to image number, time (s), load (Newtons) and percentage of the ultimate tensile strength (UTS). The acquisition of the first image was triggered after 28.40 s when the first acoustic emission hit was detected. The images presented in Figure 9 were extracted from the continuous recording at 1%, 2.7%, 10%, 76% and 98% of the ultimate tensile strength of the composite. With reference to the images presented in Figure 9, only 7%–8% of the fibres had fractured and stopped transmitting light between the first and the 2365th frame (76% of UTS). The last image that was captured just before the complete attenuation has also been included in Figure 9.

In the situation discussed previously for the un-impregnated SDOF bundles, the filament ceases to carry the applied load upon fracture (unless significant entangling of the individual filaments ensues). In the case of fibre reinforced composites, the fractured filaments continue to carry the applied load after a finite distance from the fractured-ends. Within the immediate vicinity of the fracture, the neighbouring filaments experience a higher stress concentration. This then increases the probability of fracture of the neighbouring fibres [16].

With reference to Figure 9 and image number 2460, it is tempting to speculate that the majority of the fractures occur in the top right-hand quadrant where the fibre volume fraction was lower from the onset of the tensile test. However, for such a conclusion to be justified, the SDOF bundles needs to be coherent. 

**Figure 10:**
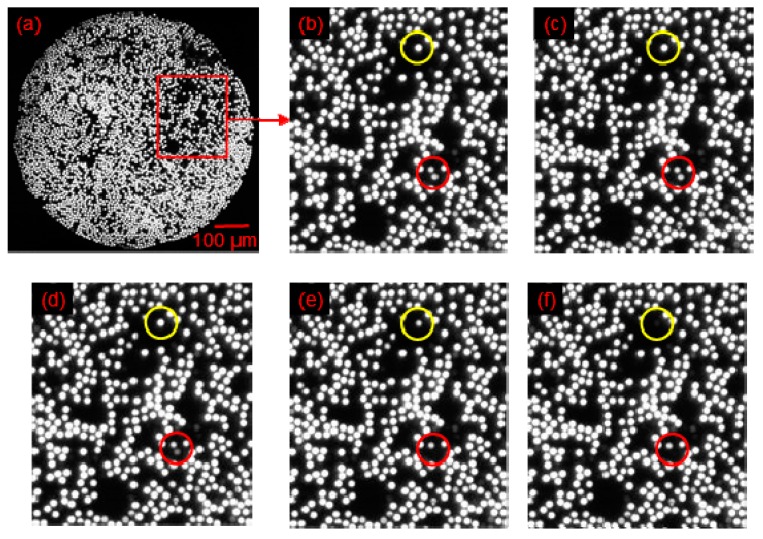
Demonstration of the sequential fibre failure in the SDOF self-sensing composite: (**a**) image captured by the high-speed camera with a highlighted section where the filaments fractured sequentially; (**b**) magnified view of the section with two fibres highlighted with red and yellow circles that are about to fracture; (**c**) image captured after 16.67 milli-seconds showing the attenuated light in the fibre shown within the red circle; (**d**) the fibre within the red-circle is about to fracture; (**e**) the fibre within the red circle was fractured whilst the light was attenuated for the fibre within the yellow-circle *i.e.*, it is about to fracture; and (**f**) both the highlighted filaments have fractured and thus stopped transmitting light.

As with the previous case with the SDOF bundle, Figure 10 shows that the image analysis routines developed were able to identify and track the imminent fracture of the filament in the composites; it is possible to identify imminent fibre fractures with an accuracy of 95% ± 2%. The series of micrographs shown in Figure 10 demonstrates the possibility of detecting progressive fibre failure in a composite test specimen. With reference to Figure 10, the image labelled (a) is the potted and polished view of the SDOF composite specimen, captured by the high-speed camera. Magnified views of a section of Figure 10a are shown in Figure 10b–f. Figure 10b–f are the consecutive frames captured by the high-speed camera at 16.67 millisecond intervals. These consecutive images were selected to demonstrate the phenomenon of sequential fibre failure. The red circles in Figure 10b–f shows that one of the fibres was fractured during tensile loading. The fibre in the red circle (Figure 10c) is about to fracture and the light is attenuated significantly as illustrated in Figure 10d. The fibre was then fractured and ceased to transmit light, as shown in Figure 10e. At exactly the same time, another fibre, highlighted by the yellow circle, fractured and the light was attenuated as shown in the same figure (Figure 10e). The fractured fibres stopped transmitting light, as illustrated in Figure 10f. In this representative example of sequential fibre failure, it has been demonstrated that it is possible to detect the imminent fibre fracture caused by the fracture of neighbouring fibres. The example presented in the preceding section could be more accurate if the fibre bundles were coherent and therefore, the fracture of the fibre highlighted by the red circle could be related to the fracture of the fibre in the yellow circle with more confidence. However, this example provides an idea of how the self-sensing concept could be used to predict and demonstrate sequential fibre failure and the onset of damage in fibre reinforced composite materials.

### 3.6. General Discussion

The self-sensing technique describe in the sections above was applied successfully to conventional E-glass bundles and composites. In this instance, a laser source was used to illuminate the E-glass bundle. The outcome of that study will be reported in a subsequent publication.

The use of reinforcing fibres as light guides was reported by Hayes *et al.* [26] and Kister *et al.* [46]. Hayes *et al.* employed quartz fibres that were embedded in a composite panel of a 16-ply carbon fibre pre-pegs composite system. Similarly, Kister *et al.* [46] used conventional E-glass fibres that were coated with epoxy or polyurethane to act as a cladding material. The coated E-glass fibres were embedded in the composite to detect damage during mechanical loading. Wang *et al.* [52] developed organo-silane sol-gel cladding formulations for E-glass fibres. They argued that since organo-silanes are used commonly as coupling agents for reinforcing glass fibres, using them as the cladding for E-glass light guides would offer chemical compatibility with the matrix. Two formulations were developed to obtain crack-free coatings: (i) tetraethoxysilane (TEOS) and polyvinyl alcohol; and (ii) acid-catalysed solutions of TEOS mixed with 3-glycidoxypropyltrimethoxysilane (GLYMO). The tensile properties of the GLYMO-coated E-glass fibres exhibited a higher displacements-to-failure. They speculated that the organo-silane cladding reduced the sensitivity of the fibres to crack initiation and propagation.

Although organo-silane-based claddings are effective in enabling light to be guided in the E-glass fibre bundles, scaling up the technology to facilitate light transmission over tens of metres is an issue; this is because of the intrinsic light attenuation characteristics of E-glass [52]. Nevertheless, it provides a unique opportunity to study the fracture behavior of E-glass fibres *in situ* as a function of surface-treatment and the processing conditions used to manufacture the composite. The authors have demonstrated that un-coated reinforcing E-glass fibres can be used to monitor the cross-linking kinetics of thermosetting resins, on the surface of E-glass fibres, using evanescent wave spectroscopy [49].

In the current work, the reinforcing SDOF were used as the sensor and thus the whole volume of the composite served as the sensing medium. This is of significance because it is impossible to predict the locality and magnitude of damage caused by events such as bird-strikes, impact damage caused by hailstones, accidental impact/collisions, dropped tools and other forms of barely visible impact damage for detecting the severity of fibre fractures in glass fibre composites. Therefore, SDOF-based self-sensing technology can provide a cost-effective method for monitoring the structural integrity of the whole composite structure. In addition to illumination of the composite from the edge (to access the end-face of the optical fibres), the feasibility of using through-thickness illumination of E-glass composites was demonstrated previously by Kister *et al.* [46]. The damaged area on the self-sensing composites was also located by means of the light bleeding or emanating from the damaged E-glass fibre. Therefore, access to a free-edge may not be necessary in every instance to illuminate the self-sensing reinforcing glass fibres. However, facilitating through-thickness illumination will depend on the light transmission characteristics of the coating on the surface of the glass fibre composite.

**Figure 11:**
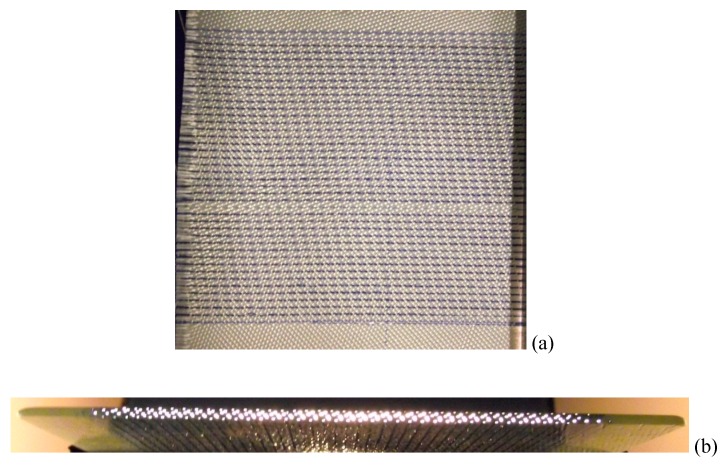
(**a**) Photograph of a co-woven SDOF/E-glass fabric; (**b**) Photograph showing the SDOFs transmitting white-light from the edge of the composite.

The scalability of the self-sensing technology was demonstrated recently by co-weaving custom-made 20-micrometer diameter optical fibres (produced by Schott AG, Mainz, Germany) into a plain-weave E-glass fabric (woven by PD-I Technologies, Sherborne, UK). Figure 11a shows a photograph of a plain weave fabric where the SDOFs were co-woven in the weft (fill) direction; the warp fibres were conventional E-glass bundles. These SDOF survived the life cycle of the weaving process from spooling, weaving, winding, surface treatment and re-spooling. Hence, it can be assumed that the robustness of the SDOF is equivalent to that of E-glass. The weave-density of the weft fibres can be controlled as desired during the weaving process. 

Figure 11b shows a composite where the SDOF preform was impregnated with an epoxy/amine resins system and processed in an autoclave. The cut-edge of the composite was illuminated with a strip (rectangular) white light source and the transmitted light via the SDOF is clearly visible in Figure 11b. In the context of self-sensing composites, the primary advantages of the SDOF over E-glass fibres are: (i) the light transmission is not limited to a few metres; and (ii) white light or laser pointers can be used to illuminate the fibres.

## 4. Conclusions

An optical fibre manufacturer was commissioned to produce the SDOF whose external diameter was 12-micrometres and this corresponds to that of commercially available E-glass fibres. Therefore, the issue of the mismatch in the diameters between the reinforcing and sensing fibres does not arise. A resin-injection method was developed and used to manufacture void-free SDOF-based composites. The method permitted the alignment and orientation of the SDOF to be maintained during impregnation and cross-linking of the matrix. Thus, it was possible to produce unidirectional composites without the presence of voids, waviness in the reinforcement or resin-rich regions. The absence of these defects enabled the failure of the reinforcement to be studied with greater confidence.

A jig was designed and built to enable one end of the SDOF to be imaged via a high-speed camera whilst the opposite end was illuminated. The SDOF bundles (unimpregnated) and the composites were tensile tested to failure whilst recording the transmitted light. Piezo-electric acoustic emission transducers were used to detect the fracture of the SDOF; the first AE signal detected was used to trigger high-speed camera.

With respect to the tensile bundle tests using un-impregnated SDOF, it was found that the majority of the fibres survive up to about 90% of the ultimate tensile strength. However, once the peak load was reached, the failure mode was not catastrophic. In the case of the SDOF composite, the attenuation in the transmitted light intensity was abrupt. Hence, this technique is ideally suited to detect the first fracture of a filament in a composite. The feasibility of identifying the imminent fracture of a filament was also demonstrated conclusively for the SDOF bundle and composite.

Scaling up of the self-sensing composite technology was demonstrated by integrating 20-micrometre diameter optical fibre bundles in the weft direction during the weaving of a plain weave E-glass fabric. The production of the custom-made SDOF and its integration into an E-glass fabric was undertaken on an industrial optical fibre drawing tower (Schott) and weaving machine (PDI-Technologies) respectively. The SDOF survived the weaving process including the various surface treatment and drying procedures that were used to apply an epoxy resin compatible “size” or coating on the fabric. The advent of self-sensing composites will enable a full life cycle analysis or cradle-to-grave assessment of glass fibre reinforced composites to be undertaken.

## Figures and Tables

**Table 1:** Summary of average mechanical properties of the SDOF composites.

Property	Average	Standard Deviation *
Elastic Modulus (GPa)	13.1	1.56
Ultimate tensile Strength (MPa)	186.50	27.18
Strain at failure (%)	1.4	0.08

* Results from six representative test specimens.

## Abbreviations

The following abbreviations are used in this manuscript:

CCD	Charge-coupled device
SDOF	Small-diameter optical fibres
SMA	Sub-Miniature Adaptor
TTL	Transistor-transistor logic
UTS	Ultimate tensile strength
